# Return-sweep saccades in oral reading

**DOI:** 10.1007/s00426-021-01610-6

**Published:** 2021-10-25

**Authors:** Victoria I. Adedeji, Martin R. Vasilev, Julie A. Kirkby, Timothy J. Slattery

**Affiliations:** Department of Psychology, Bournemouth University, Poole House, Talbot Campus, Fern Barrow Poole, Dorset, BH12 5BB UK

## Abstract

Recent research on return-sweep saccades has improved our understanding of eye movements when reading paragraphs. However, these saccades, which take our gaze from the end of one line to the start of the next line, have been studied only within the context of silent reading. Articulatory demands and the coordination of the eye–voice span (EVS) at line boundaries suggest that the execution of this saccade may be different in oral reading. We compared launch and landing positions of return-sweeps, corrective saccade probability and fixations adjacent to return-sweeps in skilled adult readers while reading paragraphs aloud and silently. Compared to silent reading, return-sweeps were launched from closer to the end of the line and landed closer to the start of the next line when reading aloud. The probability of making a corrective saccade was higher for oral reading than silent reading. These indicate that oral reading may compel readers to rely more on foveal processing at the expense of parafoveal processing. We found an interaction between reading modality and fixation type on fixation durations. The reading modality effect (i.e., increased fixation durations in oral compared to silent reading) was greater for accurate line-initial fixations and marginally greater for line-final fixations compared to intra-line fixations. This suggests that readers may use the fixations adjacent to return-sweeps as natural pause locations to modulate the EVS.

Research on eye movements during reading has been dominated by the exploration of silent reading processes. However, much can be learned from oral reading processes, especially since this is the primary modality through which children learn to read (Laubrock & Kliegl, 2015; Vorstius et al., 2014). During reading, we translate visual symbols to sounds by mapping orthography to phonological and semantic representations stored in our mental lexicon. This process of lexical activation and access has been thought to occur similarly, regardless of whether we read silently or aloud. For instance, visual word recognition processes during naming and silent reading tasks are similarly influenced by several lexical variables (Juel & Holmes, 1981; Schilling et al., 1998). However, while silent reading involves covert or inner speech, oral reading involves overt speech production (Rayner et al., 2012). This additional articulatory component in oral reading increases reading times. Compared to silent reading, eye movement patterns in oral reading are characterized by an increased number of fixations, longer fixation durations (approximately + 50 ms), higher refixation probabilities, less skipping, and shorter saccades (6–7 letters vs. 7–9 letters; Anderson & Swanson, 1937; Ashby et al., 2012; Inhoff & Radach, 2014; Kim et al., 2019; Krieber et al., 2017; Rayner, 2009; Vorstius et al., 2014).

Eye movement reading research has also been dominated by single line studies, with few experiments exploring multiline reading and the return-sweep saccades needed for such texts (see Slattery & Parker, 2019; Slattery & Vasilev, 2019). These two research tendencies (silent reading and single line reading) have resulted in a lack of research into oral reading of multiline text, especially with regards to the return-sweeps that move gaze from the end of one line to the start of the next (Parker et al., 2019a, b). Return-sweeps are still not fully understood, and recent evidence suggests that their targeting and execution may be distinct from intra-line saccades (Slattery & Vasilev, 2019). It is known that during silent reading, the first fixation on a line is longer and the last fixation on a line is shorter in duration than intra-line fixations (i.e., those that do not cross line boundaries; Abrams & Zuber, 1972; Parker et al., 2019a, b; Rayner, 1977). However, when reading aloud, the eye tends to lead the voice in the text as readers make articulatory plans (Buswell, 1920). Because of this articulatory need, and the absence of parafoveal preview information across line boundaries, reading aloud may impact the planning and execution of return-sweeps.

## Eye-movements during oral reading

Though considered a less mature way of reading, oral reading is common. Developing readers use oral reading to map written text to phonological codes and skilled adult readers may engage occasionally in oral reading when reading difficult texts (Hardyck & Petrinovich, 1970). Although less than 2% of adult respondents from a recent survey read aloud more than they read silently; reading instructions, recipes, shop signs and reading to loved ones are few ways oral reading occurs in adulthood (Duncan & Freeman, 2019). Fundamentally, oral reading processes mirror silent reading processes in many ways since eye movement measures in both reading modalities are correlated within individuals (Anderson & Swanson, 1937; Søvik et al., 2000) and across individuals of different languages (Brysbaert, 2019). However, there are differences which follow directly from the differences in the rate of silent reading which is ~ 250 words per minute (WPM) and the rate of conversational speech (~ 150 WPM). The lower rate of conversational speech reveals speed limitations of the articulatory system. Speech rates may begin to approximate silent reading rates only in trained professionals (e.g., high-speed auctioneers; Rayner et al., 2016). However, for most people and dialogues, speech rates do not approach silent reading rates due to articulatory limitations. Therefore, speech processes may constrain oculomotor processes when the two systems are simultaneously activated. As such, it is perhaps not surprising that oral reading rates lie somewhere in between conversational speech and silent reading rates (~ 180 WPM; Brysbaert, 2019).

The differences between oral and silent reading rates can be attributed to the time taken to articulate, which is often slower than the time to engage in visual and linguistic processing of text. The slower speed of articulation brings along the need to coordinate the eye and the voice through a continuous adjustment of when and where to move the eyes. Such adjustments are evident in the increase in fixation durations, refixations and regressions based on the width of the eye–voice span (EVS). The EVS is the distance between the eye and the voice and averages about 2 words or 16 characters in skilled readers (Inhoff et al., 2011; Laubrock & Kliegl, 2015; Rayner et al., 2012). However, during reading aloud the EVS changes dynamically based on moment to moment reading demands/conditions. When the EVS is too wide at the onset of fixation, the oculomotor system responds by pausing longer to allow the voice to catch up, and if the EVS remains wide at the end of the fixation, regressive saccades are more likely to be triggered (Inhoff et al., 2011; Laubrock & Kliegl, 2015). Therefore, during reading aloud, additional constraints are placed on the oculomotor system by the articulatory system which influence the decision of when and where to move the eyes. One by-product of this is that the relative influence of variables such as word frequency may be reduced during reading aloud (Huestegge, 2010; Vorstius et al., 2014). It would appear that, during oral reading, the decision to terminate a fixation may not solely be determined by word frequency but also by the phonetic characteristics of words and the continuous coordination of the eye and voice (Laubrock & Kliegl, 2015; Vorstius et al., 2014).

The yoking of the eye’s forward progress to the voice is likely accomplished by means of controlling the movement of attention which precedes saccadic eye movements (Rolfs et al., 2011; Shepherd et al., 1986; Zhao et al., 2012). Additionally, it is the pre-saccadic attentional movements that are responsible for parafoveal preview benefits (see “Discussion” below) within the E-Z Reader model (Pollatsek et al., 2006; Reichle et al., 1998). Therefore, the yoked coordination of the eye and voice may limit the amount of parafoveal processing that occurs during oral reading as attention works to hold back the forward progress of the eyes. Indeed, oral reading appears to be less influenced by parafoveal preview manipulations than silent reading. For example, Ashby et al. (2012) manipulated the availability of parafoveal information using a moving window paradigm (McConkie & Rayner, 1975). Windows of either one or three words were presented to participants while they read orally or silently whilst the other words were masked. They found that the availability of accurate parafoveal information improved reading speed in silent reading more than it did for oral reading. Similarly, Inhoff and Radach (2014) investigated the extent to which readers process words in the parafovea using the boundary paradigm (Rayner, 1975). In this paradigm, an invisible boundary is placed before a target word in the sentence to manipulate what parafoveal preview participants receive in the target word location. Once the boundary is crossed, the preview changes to the actual target word. The difference in fixation durations between a valid preview (i.e., the target itself is present) and an invalid preview (i.e., a different string of letters) is called the *preview benefit* (Rayner, 1998). The preview benefit is typically interpreted as a processing advantage that allows readers to initiate recognition processes before a word is fixated (Reichle & Reingold, 2013). Inhoff and Radach (2014) found that preview benefits were smaller in oral compared to silent reading. This further suggests that readers extract less parafoveal information during oral compared to silent reading.

### Return-sweep saccades in silent reading

Return-sweep saccades usually launch from and land some five to seven characters away from the right and left margins of successive lines, respectively (Hofmeister et al., 1999; Parker et al., 2019a, b; Rayner, 2009; Slattery & Vasilev, 2019). Furthermore, compared to intra-line saccades, return-sweep saccades are longer—typically travelling between 40 and 70 characters (Slattery & Vasilev, 2019). The landing position of a return-sweep saccade is influenced by the length of the previous line: with longer lines, landing positions shift to the right (Hofmeister et al., 1999; Parker et al., 2019a, b; Vasilev et al., 2021). Unlike intra-line saccades, where the target is assumed to be the centre of a word (known as Optimal Viewing Position [OVP]; McConkie et al., 1988), the target of the return-sweep saccade is assumed to be an area relative to the left margin that is independent of the length of the first word on a line (Slattery & Vasilev, 2019). Furthermore, return-sweep landing positions are influenced by font size information, whereby landing positions in visual angle are shifted rightwards with large compared to small font sizes (Hofmeister, 1998; Vasilev et al., 2021). This effect is independent of the length of the previous line and suggests that readers use global text characteristics to target a location on the new line that maximizes word identification processes (Vasilev et al., 2021).

Compared to intra-line saccades, return-sweep saccades are costly eye movements. Because of their length, they often tend to undershoot their target (McConkie et al., 1988). As a result, many return-sweeps are followed by a corrective saccade that takes gaze closer to the left margin of the line (Ciuffreda et al., 1976; Hofmeister et al., 1999). In fact, this occurs approximately 40–60% of the time (Slattery & Vasilev, 2019). Research shows that the probability of making a corrective saccade is determined by an increase in saccade amplitude as indexed by line length measured in degrees of visual angle where longer lines lead to more corrective saccades (Hofmeister et al., 1999; Vasilev et al., 2021). Return-sweep landing positions closer to the left margin have also been associated with fewer corrective saccades as the magnitude of undershoot error provides retinal feedback to the oculomotor system to determine whether a corrective saccade should be triggered (Hofmeister et al., 1999; Vasilev et al., 2021). Return-sweeps may also incur a large cost if they are launched too early and require a long-distance regression back to the end of the prior line, especially because line boundaries do not usually coincide with sentence boundaries (Kuperman et al., 2010).

Recent work by Parker et al. (2019a, b) has also shown that, during silent reading, children launch their return-sweeps from closer to the end of the line and land closer to the beginning of the new line compared to adults. This may occur because developing readers are less efficient in parafoveally processing words. Therefore, they may have to fixate more extreme regions of the lines to compensate for this. Furthermore, Parker et al. (2019a, b) found that children make more corrective saccades following their return-sweeps, even though they land closer to the beginning of the new line, presumably due to their need for greater foveal processing of line-initial text.

The fixations adjacent to the return-sweep give information about the distinctive feature of this long reading saccade. Line-final fixations which occur prior to launching the return-sweep saccade are characteristically shorter than intra-line fixations (Abrams & Zuber, 1972; Parker et al., 2019a, b). These fixations have been thought to be shorter in duration due to either a lack of parafoveal information at line boundaries (Rayner, 1977) or due to return-sweep planning (Kuperman et al., 2010; Mitchell et al., 2008). There are two distinct types of fixations that follow return-sweeps: (1) undersweep fixations, which are followed by a corrective leftward saccade; (2) accurate line-initial fixations, which are followed by a rightward saccade. Accurate line-initial fixations are longer than intra-line fixations, likely because they land on text that has not been processed parafoveally (Heller, 1982; Parker et al., 2017). However, undersweep fixations are shorter than other reading fixations, as they are terminated quickly in order to move the eye to a better viewing location (Abrams & Zuber, 1972; Hofmeister et al., 1999; Parker et al., 2019a, b, 2020).

### Present study

The present study explored how reading aloud influences return-sweeps compared to reading silently. This is of particular interest, because oral reading involves the coordination of the eye with the voice for fluent reading. In addition to the natural lagging feature of speech processes, readers’ eye movements are functionally ahead of the voice to obtain a sufficient view of upcoming words and to prepare phonological and articulatory codes for speech output (Buswell, 1920; Levin & Turner, 1966). However, at the end of the line, access to upcoming words (i.e., those at the start of the next line) is largely limited (Parker et al., 2017). Since return-sweeps are costly eye movements, the oculomotor system is saddled with the decision of how long to wait at the end of the line before moving to the next line so that new words can be processed and stored for articulation. Moving too early could result in an unreasonably large EVS. Wait too long, and fluent reading may be disrupted. Thus, a modulation of the EVS might impact the way return-sweeps are executed in oral reading compared to silent reading. This eye–voice coordination account where readers are monitoring the span between the voice and the eye to ensure it is neither too narrow nor too wide allows us to make predictions regarding how return-sweep spatial (launch and landing positions) and temporal measures (fixation durations adjacent to return-sweep saccades) may differ between silent and oral reading. Additionally, previous evidence has suggested that the EVS decreases towards the end of lines (Buswell, 1920; Fairbanks, 1937; Quantz, 1897), just before the return-sweep saccade would be made.

First, oral reading is characterized by shorter saccades and higher refixation rates (De Luca et al., 2013; Rayner, 2009), likely driven by the ongoing need to allow the voice to catch up with the eyes (Laubrock & Kliegl, 2015). Because of this speech lag, less attention may be given to upcoming words, thus reducing parafoveal processing compared to silent reading conditions. As a result, readers should be less likely to skip words. Therefore, words closest to the left and right margins should be more likely to receive fixations in oral reading compared to silent reading. Furthermore, if readers aim to keep the eyes from travelling far ahead of the voice through refixations, the likelihood that fixations will be nearer to the left and perhaps, right margin, becomes greater. Consequently, identical to return-sweep planning in children who have a similar need for foveal inspection (Parker et al., 2019a, b), we expect return-sweep launch and landing positions to occur closer to the right and left margin respectively, in oral reading compared to silent reading.

Second, since our expectations regarding launch and landing positions mean that readers may aim to target regions closer to the left margin, return-sweeps will be planned to travel farther in oral reading (thus increasing the susceptibility to undershoot errors). Therefore, the probability of making a corrective saccade should be greater in oral reading. This is because the farther the saccade target, the greater the probability of an undershoot (Abrams & Zuber, 1972). Furthermore, reduced parafoveal processing during reading aloud should mean that readers rely more on foveal processing and require more frequent corrective saccades to foveate line-initial words.

Third, we would expect fixation durations to be longer when reading aloud than when reading silently because the need for articulation slows down the oculomotor system. More importantly, we expect the increase in fixation durations associated with reading aloud to be greater for line-final fixations and accurate line-initial fixations than for intra-line fixations, because the system monitoring the EVS may use the fixations adjacent to the return-sweep as a natural pause to allow the voice to catch up with the eyes. Allowing the voice to catch up at the line-final fixation may help prevent a costly regression after the return-sweep saccade is made either due to a wide EVS at the end of the accurate line-initial fixation (Laubrock & Kliegl, 2015) or insufficient processing of line-final information. This wait time is particularly likely because regressions across lines are less common than regressions within lines (Ehrlich & Rayner, 1983; Rayner, 1998). This is especially plausible if fixations prior to return-sweeps function to process linguistic information (Rayner, 1977), rather than just being concerned with oculomotor planning (Mitchell et al., 2008). Such regressions at line boundaries may have a ripple effect on oral reading fluency and so the oculomotor system may attempt to prevent this from happening by modulating fixation durations adjacent to the return-sweep. Since return-sweeps launch and land about 5–7 characters from both margins (Hofmeister et al., 1999; Parker et al., 2019a, b; Rayner, 2009; Slattery & Vasilev, 2019), it follows that the intervening number of characters between return-sweep launch and landing sites may be greater than the average intra-line saccade length (10–14 vs 7–9 characters) during silent reading. This difference would lead to a wide, and potentially obvious EVS at the start of the line which the oculomotor system may aim to compensate for by increasing wait time at this location. Undersweep fixation durations, which are thought to result from oculomotor error (Hofmeister et al., 1999; Slattery & Parker, 2019), are expected to be unaffected by reading modality.

## Method

### Participants

Forty students (21 female) participated for course credits or £10 compensation. Their average age was 22 years (*SD* = 5.96 years: range 18–45 years). All participants were fluent English speakers who reported normal or corrected to normal vision and no prior diagnosis of reading disorders. All participants were naive as to the purpose of the experiment. The study was approved by Bournemouth University Research Ethics Committee (ID 26561).

### Materials and design

**Fig. 1:**
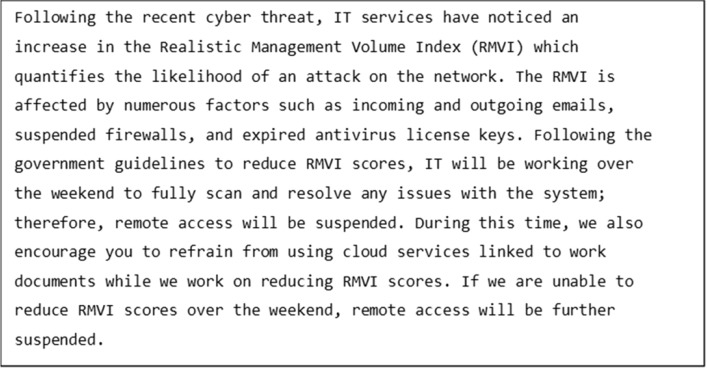
An example paragraph used in the experiment

The reading stimuli consisted of 40 multiline passages (see Fig. 1 for an example). On average, stimuli contained approximately 11 lines of text (range: 9–12). Each line had an average of 64 characters (range: 5–78). The experiment had a single factor within subject design with Reading Modality (Silent vs. Oral) as the independent variable. The data were collected as part of an experiment that examined the role of abbreviations on eye movements during oral and silent reading. In addition to Reading Modality, this within subject design included factors for target Word Frequency and target Abbreviation Type. The target word variables were not considered for the current analysis.

The assignment of conditions to sentences was counter-balanced with a full-Latin square design across all participants. The two reading modalities were blocked, and items appeared in a pseudo-random order within each block. Half of the participants read silently first, and the other half read aloud first.

### Apparatus

Eye movements were recorded with an SR Research EyeLink 1000 eye-tracker with sampling frequency of 1000 Hz. Although viewing was binocular, only the right eye was recorded (except for three participants who had their left eye recorded due to tracking problems). Participants’ head was held stable using a forehead rest without the chin rest to allow for easy articulation while reading aloud. This setting was also adopted for the silent reading block. The stimuli were presented using a Cambridge Research Systems LCD ++ monitor with a 1920 × 1080 screen resolution and a 120 Hz refresh rate. The text was formatted in an 18-point monospaced Consolas font, which appeared as black letters over a white background. The text was doubled spaced, justified to the left and presented in the middle of the screen vertically and with a 500-pixel offset horizontally. The eye-to-screen distance was 80 cm. At this distance, each letter subtended ~ 0.32° horizontally.

The experiment was programmed in MATLAB R2014a (MathWorks, 2014) using the Psychtoolbox v.3.0.11 (Brainard, 1997; Pelli, 1997) and Eyelink (Cornelissen et al., 2002) libraries. The experiment was run on a Windows 7 operating system.

### Procedure

The experiment began after participants gave written consent and verbal instructions were given. Calibration and validation accuracy were kept at < 0.40º and a recalibration was done whenever the drift check fell below this level, and after every ten trials during the experiment. Participants were asked to read the passages for comprehension either silently or aloud, depending on the instruction that appeared before the blocks. The experiment began with two practice trials during which participants read silently.

Each item was followed by a multiple-choice comprehension question with four options to ensure understanding of the text. Participants clicked the left button of the mouse to indicate they had finished reading the text and to answer the comprehension questions. The questions were either asking for specific information or the general gist of the passage. An example question for the item above (Fig. 1) is “What affects the likelihood of an attack?”, with four options: hardware, corrupt USB devices, careless formatting, and suspended firewalls. All participants were offered a short break after every ten trials.

### Data analysis

Eye movement data were pre-processed using Eye-doctor v.0.6.5 (Stracuzzi & Kinsey, 2009) to align vertical fixations on the correct line and the EMreading R package (Vasilev, 2018) software was used to extract fixation data for the analysis.

Four measures were analysed when comparing return-sweeps in oral and silent reading.

1. *Launch position*: The number of characters from the end of the line that the return-sweep saccade started.
2. *Landing position*: The number of characters from the start of the new line that the return-sweep saccade ended.
3. *Corrective saccade probability*: The likelihood that a return-sweep saccade is immediately followed by at least one additional leftward saccade.
4. *Fixation durations*: The duration of the four distinct types of fixations (Parker & Slattery, 2020; Parker et al., 2019a, b), namely:
  - Intra-line fixations: those not adjacent to a return-sweep saccade
  - Line-final fixations: those immediately prior to a return-sweep saccade
  - Accurate line-initial fixations: those immediately following a return-sweep saccade given that the fixation is followed by a rightward saccade.
  - Undersweep fixations: those immediately following a return-sweep saccade given that the fixation is followed by a leftward corrective saccade.

The data were analysed using the lme4 package v.1.1-21 (Bates et al., 2015) in R software v.4.0.3 (R Core Team, 2019). In the launch and landing position models, reading modality was a predictor and sum contrast coding was used (Oral: 1, Silent: −1). Launch distance (calculated as number of characters from the left margin) was centred with mean of 0 and was included as a covariate in the landing position and corrective saccade probability models. In the fixation duration models with Fixation type (Intra-line, Line-final, Accurate line-initial & Undersweep) as predictor, treatment contrast coding was used, where intra-line fixations were the baseline. Fixation durations were log-transformed. A full random structure with random slopes and intercepts for participants and items was initially applied (Barr et al., 2013). The maximal model was trimmed by removing higher order interaction terms and components with the least amount of variance in the random effects structure successively until convergence was achieved. The results were considered as statistically significant if the |*t*| and |*z*| values were ≥ 1.96. Cohen’s d effect sizes are also reported.

## Results

All participants achieved at least 70% on the comprehension questions, indicating that they read the passages for meaning (*M* = 85.3% *SD* = 35.4% range: 72.5–95%). Comprehension accuracy was significantly greater in oral reading than silent reading (*b* = 0.85, *SE* = 0.16, *t* = 5.3, *p* < 0.01). Fixations less than 80 ms that occurred within one-character space of a temporally adjacent fixation were combined with that fixation. Ten trials were removed due to tracking loss and accidental button presses (0.37%) and a total of 32 lines were removed from trials due to data loss (0.04%). Blinks led to the exclusion of 13.74% of fixations. Fixations less than 80 ms which were not merged with an adjacent fixation (1.41%), fixations greater than 1000 ms (0.35%), or fixations occurring outside the screen bounds (0.01%) were all discarded. These exclusions impacted the different fixation types similarly. This left a total of 84.07% of fixations for analyses (13,738 return-sweep saccades) which were evenly distributed across experimental conditions. Descriptive statistics for different measures of oral and silent reading are reported in Table 1 for general information as these are not part of the statistical analyses. Descriptive statistics related to return-sweep saccades are shown in Table 2.

**Table 1:** Mean and standard deviations (in parenthesis) for eye movement measures across oral and silent reading

Measures	Oral	Silent
Fixation duration	258 (131.51)	226 (99.85)
Single fixation duration	277 (158.15)	234 (109.61)
First fixation duration	271 (156.96)	230 (109.45)
Gaze duration	342 (225.43)	267 (150.05)
Total viewing time	414 (285.88)	324 (234.27)
Progressive saccade length^a^	6.99 (4.32)	8.66 (4.95)
Return-sweep length	57.7 (7.59)	55.1 (9.31)
Skipping probability	0.13 (0.33)	0.23 (0.42)
Regression probability	0.30 (0.46)	0.31 (0.46)
Reading rate (wpm)	156 (23.73)	241 (72.28)
Comprehension accuracy (%)	89.5 (0.31)	81.1 (0.39)

Saccade length and Return-sweep length are in number of characters

^a^Progressive saccade length excludes return-sweeps

**Table 2:** Means and standard deviations (in parenthesis) for return-sweep saccade spatial and temporal measures

Return-sweep spatial measures	Oral	Silent
Launch position	3.67 (6.15)	5.17 (6.99)
Landing position	5.69 (4.32)	6.64 (5.53)
Accurate line-initial	2.74 (3.11)	3.71 (3.90)
Undersweep	6.91 (4.15)	8.30 (5.58)
Corrective saccade probability	0.71 (0.46)	0.64 (0.48)

Return-sweep temporal measures	Fixation types		
Fixation duration (ms)	Intra-line	263 (130.79)	231 (99.16)
	Line-final	237 (135.81)	204 (105.85)
	Accurate line-initial	338 (142.60)	281 (97.08)
	Undersweep	152 (55.94)	150 (43.74)

Launch position was measured in number of characters from the end of the line, landing position in number of characters from the beginning of the line and corrective saccades as the probability of making a leftward saccade immediately following the return-sweep saccade

### Return-sweep saccade spatial measures

**Fig. 2:**
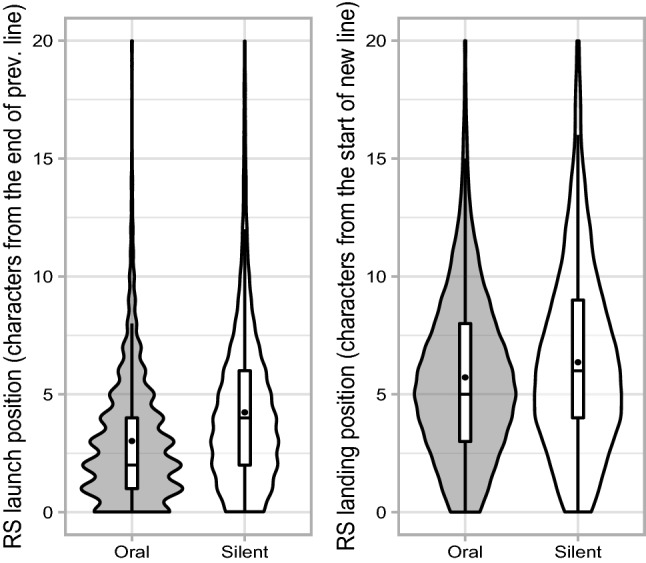
Violin plots with box plots embedded showing the distribution of return-sweep saccade launch position and landing position. Centre of box plots indicates median, while points indicate the mean

The results from the (generalized) linear mixed model ((G)LMM) are shown in Table 3 and illustrated in Fig. 2. There was a main effect of reading modality on return-sweep saccade launch position (Cohen’s *d* = −0.23, 95% CI [−0.53, 0.08]). When participants read aloud, they launched their return-sweeps from closer to the end of the line than when they read silently. Furthermore, there was a main effect of reading modality on return-sweep saccade landing position (Cohen’s *d* = −0.18, 95% CI [−0.35, −0.01]); return-sweeps landed closer to the beginning of the new line during oral reading compared to silent reading. When considering the main effect of launch distance (i.e., the distance in characters from the left margin where the return-sweep saccade is launched), landing position shifted towards the left margin, the closer from the left margin the return-sweep saccade was launched, but this was only marginally significant. Similarly, there was a marginal two-way interaction between launch distance and reading modality.

**Table 3:** (G)LMM analyses showing launch position, landing position and corrective saccades as a function of reading modality and launch distance

Fixed effects	Launch position^a^			Landing position^b^			Corrective saccades^c^		
	*b*	SE	*t*	*b*	SE	*T*	*b*	SE	*z*
Intercept	4.42	0.26	**16.85**	6.13	0.42	**14.58**	0.87	0.15	**5.87**
Modality	−0.75	0.10	**−5.81**	−0.59	0.14	**−4.16**	0.13	0.05	**2.67**
Launch distance				0.07	0.04	*1.67*	0.14	0.04	**3.36**
Modality* Launch distance				0.07	0.04	*1.66*	0.04	0.02	1.60

Statistically and marginally significant *t*/*z* values are formatted in bold and italics, respectively. Return-sweep launch distance measured from the left margin was centred to a mean of 0. Model structures for dependent measures are shown below:

^a^Launch position ~ Modality + (1 + Modality | sub) + (1 + Modality | item)

^b^Landing position ~ Modality* Launch distance + (1 + Modality +| sub) + (1 | item)

^c^Corr. saccade probability ~ Modality *Launch distance + (1 + Modality + Launch distance | sub) + (1 + Launch distance | item)

There was a main effect of reading modality on corrective saccade probability (Cohen’s *d* = 0.15, 95% CI [−0.03, 0.32]). The probability that a return-sweep saccade is followed by a leftward corrective saccade was greater for oral compared to silent reading. There was also a main effect of launch distance from the left margin, indicating that the greater the launch distance, the higher the probability of making a corrective saccade. However, there was no interaction between launch distance and reading modality on corrective saccade probability.

### Return-sweep saccade temporal measures

**Fig. 3:**
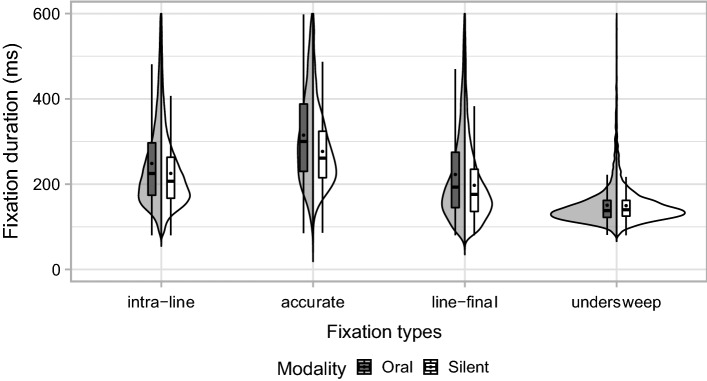
Split violin plots with box plots embedded showing the distribution of fixation durations by reading modality and fixation types. Centre of box plots indicates median while points indicate the mean. *Y*-axis limit was set at 600 ms for graphical purposes as upper bound in analyses was 1000 ms

The LMM results are shown in Table 4 and illustrated in Fig. 3. There was a main effect of reading modality as fixation durations in oral reading were generally longer than during silent reading (Cohen’s *d* = 0.26, 95% CI [0.02, 0.51]). Furthermore, the analyses revealed main effects of fixation types. Specifically, accurate line-initial fixation durations were longer (Cohen’s *d* = 0.48, 95% CI [0.17, 0.78]) while line-final (Cohen’s *d* = −0.24, 95% CI [−0.52, 0.04]) and undersweep (Cohen’s *d* = −0.96, 95% CI [−0.35, −0.57]) fixations were shorter than intra-line fixations. Crucial to our hypotheses, we found reading modality by fixation type interactions. Compared to the reading modality effect on intra-line fixations, the modality effect was marginally greater for line-final fixations. However, it was significantly greater for accurate line-initial fixations and significantly smaller for undersweep fixations. The size of the undersweep modality interaction (−0.023) nearly counters the modality main effect (0.024) indicating a lack of modality effect for undersweep fixations.

**Table 4:** LMM analyses showing fixation durations as a function of reading modality and fixation types

Fixed effects	Fixation duration^a^		
	*b*	SE	*t*
Intercept	2.3542	0.0061	**386.0729**
Modality	0.0241	0.0004	**56.7387**
Accurate line-initial fixation	0.0993	0.0026	**38.1943**
Line-final fixation	−0.0640	0.0015	**−42.2308**
Undersweep	−0.1909	0.0018	**−104.4191**
Modality: accurate line-initial fixation	0.0075	0.0026	**2.8968**
Modality: line-final fixation	0.0029	0.0015	*1.8967*
Modality: undersweep fixation	−0.0233	0.0018	**−12.7636**

Statistically and marginally significant *t*/*z* values are formatted in bold and italics, respectively

^a^log10(Fixation duration) ~ Fixation type * Modality + (1 | sub) + (1 | item)

## Discussion

The present study investigated how reading modality (silent vs. oral) affects return-sweep saccade planning and execution in adult readers. We found that readers launched their return-sweeps from closer to the right text margin and terminated it at a position that is closer to the left text margin of the next line during oral compared to silent reading. Additionally, the probability of making a corrective saccade was higher in oral reading compared to silent reading. Finally, while we replicated the robust modality effect on fixation durations, we also show for the first time that this effect was significantly greater for accurate line-initial fixations and marginally greater for line-final fixations compared to intra-line fixations.

Launch and landing positions closer to the right and left text margins, respectively, indicate that the amplitude of the return-sweep saccade is longer in oral reading than silent reading. During oral reading, articulatory constraints on the oculomotor system may limit pre-saccadic attentional shifts to parafoveal words (Pollatsek et al., 2006; Rolfs et al., 2011). This view is compatible with reduced capacity for parafoveal processing in oral reading compared to silent reading within lines (Ashby et al., 2012; Inhoff & Radach, 2014; Pan et al., 2017). Because, line boundaries are also influenced by this kind of processing, readers may foveate closer to the left and right margins to process the letters there. EVS modulation via return-sweep launch and landing sites may also occur so readers’ progressive saccades will span a similar number of characters when moving within lines and across lines. In this study, the number of new letters taken in by the visual system as evidenced by intra-line progressive saccade length was 6.99 and 8.66 characters for oral and silent reading, respectively (see Table 1). We can calculate the progressive span of return-sweep saccades by summing the number of characters to the right of its launch position with the number of characters to the left of its landing position. Doing this, we see that accurate return-sweeps have a progressive span of 6.41 and 8.88 characters for oral and silent reading, respectively (see Table 2). This indicates that roughly the same number of characters were available for processing between fixations for both intra-line and return-sweep saccades. Essentially, reading modality influenced the progressive movement of the eyes in the text similarly for intra-line and return-sweep saccades. Overall, these results are consistent with the proposition that oral reading may be a less risky reading strategy compared to silent reading, because words are skipped less often (McGowan & Reichle, 2018; McGowan et al., 2014; Rayner et al., 2006).

Early research suggested that the distance from which a return-sweep is launched may influence its landing position. Though no inferential statistics were presented, Hofmeister et al. (1999) showed that the mean launch and landing positions of return-sweeps shifted rightwards with increasing line length. We found a marginally significant launch distance effect on landing positions in this direction. Additionally, we found a marginal interaction between launch distance and reading modality. As launch site shifted to the left, so did the landing site for reading aloud but this relationship was largely absent for silent reading. Examining the scatterplot for this model revealed that this interaction may have been driven by three cases of shallow return-sweep saccades (i.e., return-sweeps that launched and landed towards the middle of lines) in the silent reading condition. Removal of those cases resulted in no interaction between reading modality and launch distance as well as a statistically significant main effect of launch distance on landing positions. Such an influence may mirror inconsistencies found with launch distance effects on return-sweeps (see Slattery & Vasilev, 2019; Vasilev et al., 2021). Considering these, more research is needed to clarify the influence of launch distance on return-sweep landing positions in skilled adult reading.

While the launch distance effect on landing position was marginal, the launch distance effect on corrective saccade probability was significant. The greater the distance from the left margin the return-sweep saccade was launched, the greater the probability of making a corrective saccade. This agrees with what has been found with previous research (Hofmeister et al., 1999; Slattery & Vasilev, 2019; Vasilev et al., 2021) and reflects the fact that undershoots are increasingly likely to occur the farther away the eyes are from the saccade’s target location.

Reading modality also significantly influenced corrective saccade probability, which was higher in oral compared to silent reading. This result is similar to the finding that children are more likely to initiate such corrective leftward saccades than adults, presumably to enable more precise foveal encoding of words at line extremities (Parker et al., 2019a, b). Despite the tendency to foveate closer to the left margin at the start of a new line when reading aloud, our readers nevertheless made more corrective leftward saccades in this condition. This may be explained by assuming that readers target an area closer to the left margin when reading aloud to enable foveal processing of line-initial characters, resulting in longer intended saccades and, therefore, increased saccadic error (McConkie et al., 1988). This increased saccadic error would then result in an increased need for corrective leftward saccades (Slattery et al., in preparation). The increase in corrective saccade probability may also modulate the eye–voice span as readers may be more likely to initiate a corrective saccade if the EVS at the end of the last fixation on the line was wide. In this way, corrective saccades may serve a similar function as regressions in modulating the EVS (Laubrock & Kliegl, 2015).

A considerable amount of work has shown that fixation durations are longer when reading aloud compared to when reading silently (Anderson & Swanson, 1937; Krieber et al., 2017; Laubrock & Kliegl, 2015; Rayner, 2009; Vorstius et al., 2014). Our results are clearly consistent with these studies. This suggests that the oculomotor system may delay progressive saccade generation to prevent a wide EVS (Inhoff et al., 2011; Laubrock & Kliegl, 2015). As in previous return-sweep studies, we found that compared to intra-line fixations, line-final and undersweep fixations were shorter and accurate line-initial fixations were longer (Abrams & Zuber, 1972; Heller, 1982; Hofmeister et al., 1999; Parker et al., 2019a, b, 2020; Rayner, 1977). However, what remained unknown was how the reading modality effect may differentially influence fixations adjacent to the return-sweep. We hypothesized that the increase in fixation durations in oral compared to silent reading would be greater for line-final fixations and accurate line-initial fixations compared to intra-line fixations due to EVS coordination at line boundaries. As expected, the reading aloud cost was significantly greater for accurate line-initial fixation durations (57 ms) and marginally greater for line-final fixations (33 ms) during oral reading when compared to intra-line fixations (32 ms). The implication of this finding is that the oral reading cost, while pervasive throughout the text being read, was greater around return-sweeps (particularly after them), suggesting that these fixations offer a suitable opportunity for EVS modulation.

The fixations intervening between the return-sweep and corrective saccade has been called undersweep fixations (Parker et al., 2017, 2020). Whether or not these fixations are involved in ongoing linguistic processing has been a subject of recent research (Parker et al., 2020; Slattery & Parker, 2019). Our results reveal that the reading modality effect was absent for undersweep fixations (2 ms). This is in line with the proposition that these fixations result from oculomotor error (Hofmeister et al., 1999; Slattery & Parker, 2019). The implication of this finding is that undersweep fixations are not sensitive to the additional articulatory demands of oral reading nor the modulation of the EVS.

In summary, the fixation duration results suggest that the fixations around return-sweeps (line-final and accurate line-initial fixations) may offer a natural pause in the acquisition of new linguistic information and may modulate the EVS during oral paragraph reading. The coordination of the eye and voice causes a reliance on foveal processing rather than parafoveal processing in oral reading which reflected in the launch position, landing position and corrective saccade probability results. It is apparent that oral reading imposes restrictions on eye movements not only because of the time required to articulate words but also the time allocated to articulatory pauses (Godde et al., 2021). These pauses are essential not just for intelligible speech production (Quantz, 1897) but also physiologically, articulation occurs mostly during periods of exhalation and not inhalation (Huey, 1908). Paragraph reading involves the integration of meaning across multiple sentences and lines (Cook & Wei, 2019). While longer fixation pauses are made at sentence boundaries due to sentence wrap up effects (Kuperman et al., 2010; Tiffin-Richards & Schroeder, 2018), intermittent pauses are also made by the articulatory system at phrase units, sentence boundaries and punctuation marks during oral reading. In addition to this, we propose that, during oral reading, the oculomotor system may also use line boundaries, as pause points to ensure a reasonable EVS. Although, it could also be that reading tasks that generally require more attention to word processing, such as oral reading, may cause saccade generation at the start and end of lines to be delayed. To explore the plausibility of this and increase our understanding of return-sweeps, future research may seek to compare the influence of different reading tasks varying in cognitive demands (e.g., skimming, proofreading or reading while listening; Valentini et al., in preparation), on return-sweep saccade execution and targeting.

## Data Availability

The datasets generated and analysed during the current study are available in the Open Science Framework (OSF) repository, https://osf.io/ym76z/.

## References

[CR1] Abrams SG, Zuber BL (1972). Some temporal characteristics of information processing during reading. Reading Research Quarterly.

[CR2] Anderson IH, Swanson DE (1937). Common factors in eye movements in silent and oral reading. Psychological Monographs.

[CR3] Ashby J, Yang J, Evans KH, Rayner K (2012). Eye movements and the perceptual span in silent and oral reading. Attention, Perception & Psychophysics.

[CR4] Barr DJ, Levy R, Scheepers C, Tily HJ (2013). Random effects structure for confirmatory hypothesis testing: Keep it maximal. Journal of Memory and Language.

[CR5] Bates D, Mächler M, Bolker B, Walker S (2015). Fitting linear mixed-effects models using lme4. Journal of Statistical Software.

[CR6] Brainard DH (1997). The psychophysics toolbox. Spatial Vision.

[CR7] Brysbaert M (2019). How many words do we read per minute? A review and meta-analysis of reading rate. Journal of Memory and Language.

[CR8] Buswell, G. T. (1920). An experimental study of the eye voice span in reading. https://archive.org/details/experismentalstud00busw/page/n8.

[CR9] Ciuffreda KJ, Bahill AT, Kenyon RV, Stark L (1976). Eye movements during reading: Case reports. American Journal of Optometry and Physiological Optics.

[CR10] Cook AE, Wei W (2019). What can eye movements tell us about higher level comprehension?. Vision (basel).

[CR11] Cornelissen FW, Peters EM, Palmer J (2002). The Eyelink Toolbox: Eye tracking with MATLAB and the Psychophysics Toolbox. Behavior Research Methods, Instruments, & Computers.

[CR12] De Luca M, Pontillo M, Primativo S, Spinelli D, Zoccolotti P (2013). The eye-voice lead during oral reading in developmental dyslexia. Frontiers in Human Neuroscience.

[CR13] Duncan S, Freeman M (2019). Adults Reading aloud: A survey of contemporary practices in Britain. British Journal of Educational Studies.

[CR14] Ehrlich K, Rayner K (1983). Pronoun assignment and semantic integration during reading: Eye movements and immediacy of processing. Journal of Verbal Learning and Verbal Behaviour.

[CR15] Fairbanks G (1937). The relation between eye movements and voice in the oral reading of good and poor silent readers. Psychological Monographs.

[CR16] Godde E, Bailly G, Bosse M-L (2021). Pausing and breathing while reading aloud: Development from 2nd to 7th grade in French speaking children. Reading and Writing.

[CR17] Hardyck CD, Petrinovich LF (1970). Subvocal speech and comprehension level as a function of the difficulty level of reading material. Journal of Verbal Learning and Verbal Behaviour.

[CR18] Heller D, Groner R, Fraisse P (1982). Eye movements in reading. Cognition and eye-movements.

[CR19] Hofmeister, J. (1998). *Über Korrektursakkaden beim Lesen von Texten und bei leseähnlichen Aufgaben*. Shaker Verlag.

[CR20] Hofmeister, J., Heller, D., & Radach, R. (Eds.). (1999). *The return sweep in reading*. Springer. 10.1007/978-1-4757-3054-8_49.

[CR21] Huestegge L (2010). Effects of vowel length on gaze durations in silent and oral reading. Journal of Eye Movement Research.

[CR22] Huey, E. B. (1908). *The psychology and pedagogy of reading*. The Macmillian Company. https://archive.org/details/psychologypedago00hueyuoft/page/n3/mode/2up?ref=ol&view=theater.

[CR23] Inhoff AW, Radach R (2014). Parafoveal preview benefits during silent and oral reading: Testing the parafoveal information extraction hypothesis. Visual Cognition.

[CR24] Inhoff AW, Solomon M, Radach R, Seymour BA (2011). Temporal dynamics of the eye–voice span and eye movement control during oral reading. Journal of Cognitive Psychology.

[CR25] Juel C, Holmes B (1981). Oral and silent reading of sentences. Reading Research Quarterly.

[CR26] Kim Y-SG, Petscher Y, Vorstius C (2019). Unpacking eye movements during oral and silent reading and their relations to reading proficiency in beginning readers. Contemporary Educational Psychology.

[CR27] Krieber M, Bartl-Pokorny KD, Pokorny FB, Zhang D, Landerl K, Korner C, Pernkopf F, Pock T, Einspieler C, Marschik PB (2017). Eye movements during silent and oral reading in a regular orthography: Basic characteristics and correlations with childhood cognitive abilities and adolescent reading skills. PLoS ONE.

[CR28] Kuperman V, Dambacher M, Nuthmann A, Kliegl R (2010). The effect of word position on eye-movements in sentence and paragraph reading. Quarterly Journal of Experimental Psycholology (hove).

[CR29] Laubrock J, Kliegl R (2015). The eye-voice span during reading aloud. Frontiers in Psychology.

[CR30] Levin, H., & Turner, E. A. (1966). Sentence Structure and eye voice span. *Studies in Oral Reading*, *9*. https://eric.ed.gov/?id=ED011957.

[CR01] MathWorks (2014). Matlab R2014a [Computer software].

[CR31] McConkie GW, Kerr PW, Reddix MD, Zola D (1988). Eye movement control during reading: I. The location of initial eye fixations on words. Vision Research.

[CR32] McConkie GW, Rayner K (1975). The span of effective stimulus during fixation in reading. Perception & Psychophysics.

[CR33] McGowan VA, Reichle ED (2018). The “risky” reading strategy revisited: New simulations using E-Z Reader. Quarterly Journal of Experimental Psycholology (hove).

[CR34] McGowan VA, White SJ, Paterson KB (2014). The effects of interword spacing on the eye movements of young and older readers. Journal of Cognitive Psychology.

[CR35] Mitchell DC, Shen X, Green MJ, Hodgson TL (2008). Accounting for regressive eye-movements in models of sentence processing: A reappraisal of the Selective Reanalysis hypothesis. Journal of Memory and Language.

[CR36] Pan J, Yan M, Laubrock J (2017). Perceptual span in oral reading: The case of Chinese. Scientific Studies of Reading.

[CR37] Parker AJ, Kirkby JA, Slattery TJ (2017). Predictability effects during reading in the absence of parafoveal preview. Journal of Cognitive Psychology.

[CR38] Parker AJ, Kirkby JA, Slattery TJ (2020). Undersweep fixations during reading in adults and children. Journal of Experimental Child Psychology.

[CR39] Parker AJ, Nikolova M, Slattery TJ, Liversedge SP, Kirkby JA (2019). Binocular coordination and return-sweep saccades among skilled adult readers. Journal of Vision.

[CR40] Parker AJ, Slattery TJ (2020). Spelling ability influences early letter encoding during reading: Evidence from return-sweep eye movements. Quarterly Journal of Experimental Psychology (hove).

[CR41] Parker AJ, Slattery TJ, Kirkby JA (2019). Return-sweep saccades during reading in adults and children. Vision Research.

[CR42] Pelli DG (1997). The VideoToolbox software for visual psychophysics: Transforming numbers into movies. Spatial Vision.

[CR43] Pollatsek A, Reichle ED, Rayner K (2006). Tests of the EZ Reader model: Exploring the interface between cognition and eye-movement control. Cognitive Psychology.

[CR44] Quantz, J. O. (1897). Problems in the psychology of reading. *The Psychological Review*, *2*, i-51. 10.1037/h0092985.

[CR45] Rayner K (1977). Visual attention in reading: Eye movements reflect cognitive processes. Memory & Cognition.

[CR46] Rayner K (1998). Eye movements in reading and information processing: 20 years of research. Psychological Bulletin.

[CR47] Rayner K (2009). Eye movements and attention in reading, scene perception, and visual search. Quarterly Journal of Experimental Psychology (hove).

[CR48] Rayner K, Pollatsek A, Ashby J, Clifton C (2012). Psychology of reading.

[CR49] Rayner K, Reichle ED, Stroud MJ, Williams CC, Pollatsek A (2006). The effect of word frequency, word predictability, and font difficulty on the eye movements of young and older readers. Psychology and Aging.

[CR50] Rayner K, Schotter ER, Masson ME, Potter MC, Treiman R (2016). So much to read, so little time: How do we read, and can speed reading help?. Psychological Science in the Public Interest.

[CR03] R Core Team. (2019). *R: A language and environment for statistical computing (3.53)*. R Foundation for Statistical Computing. http://www.r-project.org/.

[CR52] Reichle ED, Pollatsek A, Fisher DL, Rayner K (1998). Toward a model of eye movement control in reading. Psychological Review.

[CR51] Reichle, E., & Reingold, E. (2013). Neurophysiological constraints on the eye-mind link [10.3389/fnhum.2013.00361]. *Frontiers in Human Neuroscience, 7*, 361. 10.3389/fnhum.2013.00361. 10.3389/fnhum.2013.00361 PMC3710954 23874281

[CR53] Rolfs M, Jonikaitis D, Deubel H, Cavanagh P (2011). Predictive remapping of attention across eye movements. Nature Neuroscience.

[CR02] Stracuzzi, D. J., & Kinsey, J. D. (2009). *EyeDoctor (Version 0.6.5) [Computer Software] (0.6.5)*. http://blogs.umass.edu/eyelab.

[CR54] Schilling HEH, Rayner K, Chumbley J (1998). Comparing naming, lexical decision, and eye fixation times: Word frequency effects and individual differences. Memory & Cognition.

[CR55] Shepherd M, Findlay JM, Hockey RJ (1986). The relationship between eye movements and spatial attention. The Quarterly Journal of Experimental Psychology.

[CR04] Slattery, T. J., Vasilev, M. R., & Parker, A. J. (in preparation). An examination of corrective saccades during reading: Evidence from return-sweeps.

[CR56] Slattery TJ, Parker AJ (2019). Return sweeps in reading: Processing implications of undersweep-fixations. Psychonomic Bulletin & Review.

[CR57] Slattery TJ, Vasilev MR (2019). An eye-movement exploration into return-sweep targeting during reading. Attention, Perception & Psychophysics.

[CR58] Søvik N, Arntzen O, Samuelstuen M (2000). Eye-movement parameters and reading speed: A study of oral and silent reading performances of twelve-year-old children. Reading and Writing.

[CR59] Tiffin-Richards SP, Schroeder S (2018). The development of wrap-up processes in text reading: A study of children's eye movements. Journal of Experimental Psychology: Learning, Memory & Cognition.

[CR07] Vasilev, M. R. (2018). *EMreading: Automatic pre-processing of eye-movement reading data*. R package version 0.0.1.2. https://github.com/martin-vasilev/EMreading.

[CR05] Valentini, A., Pye, R. E., Houston-Price, C., Ricketts, J. & Kirkby, J. A. (in preparation). Online processing shows advantages of bimodal listening-while-reading for vocabulary learning: an eye tracking study.

[CR60] Vasilev MR, Adedeji VI, Laursen C, Budka M, Slattery TJ (2021). Do readers use character information when programming return-sweep saccades?. Vision Research.

[CR61] Vorstius C, Radach R, Lonigan CJ (2014). Eye movements in developing readers: A comparison of silent and oral sentence reading. Visual Cognition.

[CR62] Zhao M, Gersch TM, Schnitzer BS, Dosher BA, Kowler E (2012). Eye movements and attention: The role of pre-saccadic shifts of attention in perception, memory and the control of saccades. Vision Research.

